# Variations in the Botulinum Neurotoxin Binding Domain and the Potential for Novel Therapeutics

**DOI:** 10.3390/toxins10100421

**Published:** 2018-10-20

**Authors:** Jonathan R. Davies, Sai Man Liu, K. Ravi Acharya

**Affiliations:** 1 Department of Biology and Biochemistry, University of Bath, Bath BA2 7AY, UK; jd769@bath.ac.uk; 2 Ipsen Bioinnovation Limited, Abingdon OX14 4RY, UK; sai.man.liu@ipsen.com

**Keywords:** botulinum neurotoxins, binding domain, ganglioside, SV2, synaptotagmin, neurones

## Abstract

Botulinum neurotoxins (BoNTs) are categorised into immunologically distinct serotypes BoNT/A to /G). Each serotype can also be further divided into subtypes based on differences in amino acid sequence. BoNTs are ~150 kDa proteins comprised of three major functional domains: an N-terminal zinc metalloprotease light chain (LC), a translocation domain (H_N_), and a binding domain (H_C_). The H_C_ is responsible for targeting the BoNT to the neuronal cell membrane, and each serotype has evolved to bind via different mechanisms to different target receptors. Most structural characterisations to date have focussed on the first identified subtype within each serotype (e.g., BoNT/A1). Subtype differences within BoNT serotypes can affect intoxication, displaying different botulism symptoms in vivo, and less emphasis has been placed on investigating these variants. This review outlines the receptors for each BoNT serotype and describes the basis for the highly specific targeting of neuronal cell membranes. Understanding receptor binding is of vital importance, not only for the generation of novel therapeutics but also for understanding how best to protect from intoxication.

## 1. Botulinum Neurotoxins

Botulinum neurotoxins (BoNTs) are produced mainly by *Clostridium botulinum*, under anaerobic conditions [1], and are the causative agent of botulism—a rare disease that can lead to paralysis and eventually death. The *C. botulinum* taxon can be divided into four groups (I, II, III, and IV), based on phenotypic differences between the bacteria [2]. *C. botulinum* group I (proteolytic) and group II (non-proteolytic) are mostly responsible for human botulism, whereas *C. botulinum* group III is responsible for botulism in other animal species, and *C. botulinum* group IV does not appear to cause botulism [2,3]. Across these phenotypes, a range of serologically distinct BoNTs have been identified and classified within different serotypes. Until recently, all BoNTs have been categorised into one of seven serotypes ranging from BoNT/A to BoNT/G. The recent identification of novel BoNTs and BoNT-like proteins, which are not neutralisable by existing anti-sera, has lead to classification that does not currently continue from the classical nomenclature (e.g., BoNT/X). Some *C. botulinum* strains have also been identified which express more than one serotype and/or chimeric neurotoxins (e.g., BoNT/CD and BoNT/DC).

**Figure 1:**
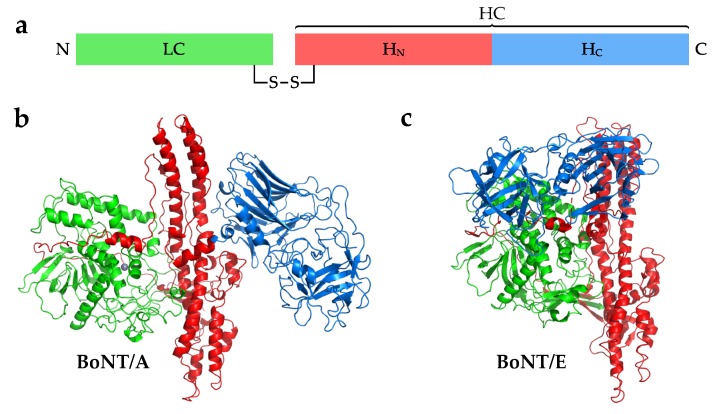
(**a**) Schematic of botulinum neurotoxin (BoNT) domain organisation. The crystal structures of (**b**) BoNT/A [10] and (**c**) BoNT/E [11] show different orientations of the receptor-binding domain (H_C_) with respect to the rest of the molecule. H_N_: translocation domain; HC: heavy chain; LC: light chain.

Each BoNT is expressed as a single polypeptide chain of ~150 kDa (Figure 1a), after which it is cleaved post-translationally by a protease to yield an active di-chain molecule consisting of a ~50 kDa light chain (LC) and a ~100 kDa heavy chain (HC) linked by a disulphide bond. Some serotypes are cleaved into a di-chain by an endogenous host protease, while others may be cleaved in the target organism [4,5]. For example, BoNT/A purified from *C. botulinum* culture after 8 h is mostly as a single-chain peptide, but when purified from a 96 h culture it is in the nicked di-chain form [6]. The clostridial protease responsible for cleaving BoNT/A has been partially characterised, but not yet identified [7]. The disulphide bond connecting the LC and HC is vital for the mechanism of intoxication [8,9]. The LC possesses a zinc endopeptidase from the M27 family of peptidases, while the HC consists of two domains—a translocation domain (H_N_) and a receptor-binding domain (H_C_). The H_C_ domain can be further divided into two distinct folds: a carboxyl-terminal β-trefoil (H_CC_) with an amino-terminal lectin-like jelly roll (H_CN_). The crystal structure of BoNT/A1 shows that the domains are arranged in a “butterfly” arrangement where the LC and H_C_ are the “wings” attached to the central H_N_ “body” (Figure 1b) [10]. Each domain appears to be structurally and mechanistically distinct from one another, with the exception of a large loop, termed the belt, which wraps around the LC. The structure of BoNT/B follows the same arrangement of BoNT/A, whereas the structure of BoNT/E appears to adopt a “closed wing” compact conformation where the H_C_ is rotated around the H_N_ and LC (Figure 1c) [11,12]. The H_C_ domain is responsible for targeting the protein to the neuronal membrane by binding to receptors present on the cell surface. All classical BoNT serotypes (/A to /G) bind to one or more polysialogangliosides, and most also bind to a protein receptor and together form a dual-receptor complex [13,14].

Polysialogangliosides consist of a hydrophilic complex polysaccharide with many sialic acid residues, bound to a hydrophobic ceramide tail. Different forms of these gangliosides can be found embedded in the cell membrane with the various sugar moieties displayed on the cell surface. The most common examples found on neuronal membranes include GT1b, GD1a, GD1b, and GM1. Two types of BoNT protein receptors have been identified to date: three isoforms of synaptic vesicle glycoprotein 2 (SV2A-C) and two isoforms of synaptotagmin (SytI-II). Both types are involved with the regulated secretion of neurotransmitter from synaptic vesicles [15,16]. SV2A-C contribute to the modulation of exocytosis, although their exact role is yet to be determined, while SytI and SytII are calcium-sensitive membrane proteins also involved in exocytosis [15,17,18,19]. Their involvement in synaptic vesicle endocytosis also requires them to be recycled back into the cell through endocytosis, making them excellent targets for BoNTs.

**Figure 2:**
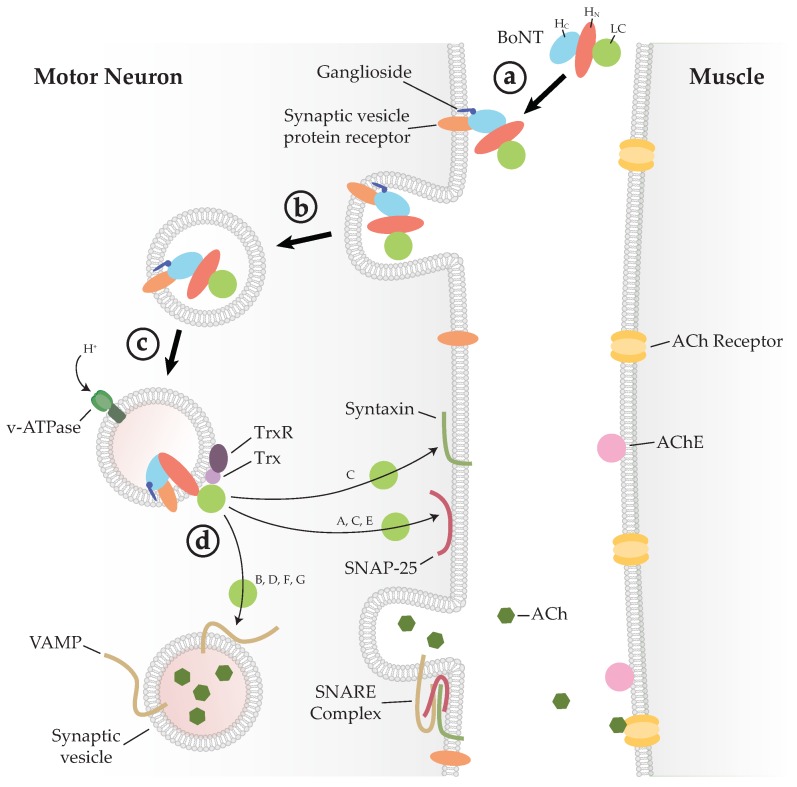
Mechanism of BoNT intoxication. (**a**) BoNT binds the neuronal cell membrane through a dual-receptor complex. (**b**) The BoNT–receptor complex is endocytosed and enclosed within a vesicle. (**c**) Acidification of the endocytic vesicle causes a conformational change, allowing the LC to be translocated through the membrane. (**d**) Thioredoxin (Trx), bound to the vesicle membrane, catalyses the reduction of a disulphide bond that releases the LC into the cytoplasm where it can cleave its soluble N-ethylmaleimide-sensitive factor attachment protein receptor (SNARE) substrate. Cleavage of any one of the SNARE proteins of the SNARE complex inhibits membrane fusion and acetylcholine (ACh) release, thus stopping muscle contraction. ACh: acetylcholine; AChE: acetylcholine esterase; Trx: thioredoxin; TrxR: thioredoxin reductase.

Once the BoNT has bound to its target receptors, it is internalised into a vesicle by endocytosis. The vesicle then matures into an endosome, and proton pumps reduce the internal pH which may cause the BoNT to undergo a conformational change. The exact mechanism of translocation is still not well understood, but it is proposed that the H_N_ forms a pore through which a partially unfolded LC passes into the neuronal cytosol [20,21,22]. The LC remains bound to the H_N_ on the cytosolic side due to a single disulphide bond, and requires host protein thioredoxin (Trx) and its partner thioredoxin reductase (TrxR) to release the LC (Figure 2). Disulphide cleavage is essential to intoxication, and inhibition of Trx is sufficient to block the LC release [23,24]. The free LC is then able to cleave a soluble N-ethylmaleimide-sensitive factor attachment protein receptor (SNARE), which prevents vesicle–plasma membrane fusion, thus inhibiting exocytosis and release of acetylcholine, causing flaccid paralysis.

## 2. Receptor-Binding Domain Variation

### 2.1. BoNT/A

Within the BoNT/A serotype there are currently eight subtypes BoNT/A1 to /A8 which differ by between 3% and 16% at the amino acid level (Table 1). The most thoroughly characterised BoNT subtype is BoNT/A1—this is in part due to its use as a therapeutic for several conditions such as spasticity, dystonias, and glabellar lines [25,26]. The carboxyl-terminal half of the BoNT H_C_ domain (H_CC_) contains the peptide motif, H...SxWY…G, which constitutes the core of the ganglioside-binding site (GBS) [27]. In contrast to the dual ganglioside binding sites identified on the related TeNT H_C_, the GBS of BoNT/A1 can only bind one ganglioside at a time [28], but is capable of recognising more than one type, specifically GT1b, GD1a, and to a lesser extent GM1 [29,30]. The exact interactions between ganglioside and the GBS were first determined from the crystal structure of the H_C_ domain in complex with GT1b [31] (PDB ID: 2VU9). This revealed extensive hydrogen-bonding with four of the seven individual monosaccharides within GT1b. Depletion of gangliosides in neuroblastoma cells completely prevents entry of BoNT/A1 [29]. However, gangliosides alone do not mediate cellular entry—for this, BoNT/A also requires the protein receptor SV2 [32,33]. Of the three SV2 isoforms found in humans, BoNT/A1 has the greatest affinity for SV2C [32]. BoNT/A1 binds specifically to the luminal domain 4 of SV2 (SV2-LD4) via direct backbone–backbone interactions between a β-strand of SV2-LD4 and a β-strand of BoNT H_C_ [34], and also through interactions with an N559-linked glycan [35]. The significance of the latter is highlighted by the inability of BoNT/A1 to bind to bacterially-expressed (i.e., non-glycosylated) SV2A or SV2B, and a reduced affinity for non-glycosylated SV2C [33,36]. The crystal structure of BoNT/A1–gSV2C-LD4 revealed a large range of interactions between the H_C_ and the SV2 glycan, which extended away from the backbone–backbone interactions, almost doubling the contact surface area [37].

The other subtypes of BoNT/A are predicted to bind the same receptors as BoNT/A1 due to their high sequence identity between the binding domains (Table 1). The crystal structure of the BoNT/A2 H_C_ domain in complex with a non-glycosylated SV2C-LD4 showed that the binding mode is conserved, and despite some residue differences, it still binds SV2C [38,39]. F563 of SV2-LD4 forms a π-stacking interaction with R1156 of BoNT/A1, while a glutamic acid residue in BoNT/A2 (E1156) causes F563 to adopt a different conformation and the BoNT instead interacts directly with H564 of SV2C-LD4. Such mutations indicate flexibility with respect to the backbone–backbone interaction of SV2, which may also be tolerated due to extra interactions with the N-linked glycan [37]. BoNT/A2 has also been shown to have a higher affinity for gangliosides than BoNT/A1, although the interactions mediating this difference have not yet been identified [40]. The crystal structures of the BoNT/A3 and /A4 H_C_ domains suggest a similar mode of interaction to ganglioside compared to BoNT/A1—the GBS of the former shows a potential loss of a hydrogen bond to one of the terminal sialic acids due to a difference in amino acid (phenylalanine rather than a tyrosine), whereas the latter is conformationally conserved [41]. With regard to the SV2 binding site, both structures show slight differences in conformation compared to that of BoNT/A1 due to differences in the primary sequence. Whether this will affect interactions with the SV2 glycan is yet to be determined. Despite high sequence identity between BoNT/A subtypes, significant differences in their intoxication properties have been identified. For example, BoNT/A2 is more potent in neuronal cells than BoNT/A1, possibly due to faster cell entry [42,43,44], and BoNT/A4 has been reported to be three orders of magnitude less potent than BoNT/A1 [44]. It is difficult to attribute these differences to just interactions between the H_C_ and receptors, and is instead likely to be as a result of contributions from the H_C_, H_N_, and LC combined. Uncovering the subtle structural changes resulting from sequence variation which may affect receptor affinity requires further work through structural studies of individual H_C_ domains and their complexes with receptors.

### 2.2. BoNT/B

There are currently eight subtypes within the BoNT/B serotype (BoNT/B1 to /B8), and they differ by between 1.5% and 7% at the amino acid level (Table 2) [54]. Although the crystal structure of BoNT/B1 exists in an open conformation similar to that of BoNT/A [11], the BoNT/B serotype targets a different protein receptor on the neuronal cell membrane, namely SytI or SytII [55,56,57,58]. Crystal structures of the HC domain from BoNT/B in complex with murine SytII revealed the high specificity of the binding interface where the SytII peptide forms a helix and binds to a hydrophobic groove via six hydrophobic residues [59,60]. Interestingly, BoNT/B displays a much lower affinity toward human SytII than murine SytII due to a single mutation at residue 54-Phe in rodents and Leu in humans [59,61,62]. Considering that SytII is more abundant on human motor neurons than SytI, a significantly larger dose of BoNT/B needs to be administered in order to achieve a similar therapeutic effect to that of BoNT/A. To overcome this issue, the BoNT/B binding domain has been engineered (E1191M,S1199Y) to increase its binding affinity—this molecule showed an 11-fold higher functional efficacy in human cells compared to wild-type BoNT/B1 [63]. BoNT/B is only capable of entering cells once it has bound to both its synaptotagmin receptor and its ganglioside receptor, either GT1b or GD1a [28,55,64]. The crystal structure of the BoNT/B1 binding domain in complex with both SytII and GD1a show strong interactions with the Sia5 moiety [65]. Although there is no direct contact between SytII and GD1a, there is some evidence that each can influence binding to the other, possibly due the spatial arrangement of both binding sites [66]. In addition to the dual receptors, BoNT/B has been reported to interact directly with the cell membrane through an exposed hydrophobic loop (“lipid-binding loop”) located between the ganglioside and Syt binding sites on the H_C_ [67].

### 2.3. BoNT/C

BoNT/C (specifically known as BoNT/C1) is predominantly associated with botulism in animals rather than humans [2,73]. There are no subtypes of the BoNT/C serotype—only two distinct protein sequences have been identified to-date (UniProtKB: P18640 [74], Q93HT3 [75]) which share 99.9% identity. Perhaps confusingly, there are two other botulinum toxins called “C2 toxin” and “C3” which are not “traditional” neurotoxins, but rather refer to different gene products—a binary AB toxin and an exoenzyme, respectively [76,77,78]. The mechanisms of cell-binding is of great interest because unlike the majority other BoNTs, no protein receptor for BoNT/C1 has yet been identified [79,80]. Interestingly, while the conserved SxWY ganglioside-binding motif is absent from the H_C_ domain, BoNT/C1 is still able to bind gangliosides [81]. Indeed, an extended hydrophobic loop termed the “ganglioside binding loop” (GBL) was reported to be essential for neuronal binding, but the specific interactions have yet to be determined [80]. Crystal structures of the BoNT/C1 H_C_ domain in complex with sialic acid revealed two potential binding sites that are independent of the GBS identified in other BoNTs [82,83].

### 2.4. BoNT/D

Like BoNT/C, there are no subtypes of BoNT/D, of which there are multiple sequences that share a primary sequence identity of >96%. BoNT/D appears to recognise all three isoforms of SV2 [81,82]. Cells lacking SV2 do not get intoxicated by BoNT/D, but this can be restored by the expression of any of the three SV2 isoforms (SV2A, B, C) [84]. It was further demonstrated that SV2A/B knockout neurones displaying a chimeric form of SV2-LD4 (SV2A, B, or C) alone were unable to mediate BoNT/D entry despite rescuing intoxication for BoNT/A and /E. Mutation of the N537 N-linked glycosylation site also had no effect on BoNT/D entry, despite blocking entry to BoNT/E [84]. This suggests that the SV2 receptor-binding domain in BoNT/D may be distinct from other SV2-interacting BoNTs such as BoNT/A. Gangliosides are also required for BoNT/D cell entry [85], however like BoNT/C, BoNT/D does not contain an SxWY motif in the GBS, although the site is still able to recognise gangliosides [86]. It is also proposed to contain a second binding site termed Sia-1, since mutation of this site results in reduced ganglioside binding [87].

### 2.5. BoNT/E

There are currently twelve known BoNT/E subtypes (BoNT/E1 to /E12) whose amino acid identities vary by up to 12% (Table 3). The protein receptor for BoNT/E is SV2, although only isoforms SV2B and SV2C are capable of mediating entry [36,88], and in the presence of gangliosides [89]. The SxWy motif is conserved in the BoNT/E GBS, and direct binding of GT1b has been observed [90]. No crystal structures of BoNT/E in complex with receptor or ganglioside have yet been solved. Therefore, the precise molecular basis of their interactions have yet to be determined. The native crystal structure of BoNT/E has been solved, and it reveals a conformation that is significantly different from that of BoNT/A and BoNT/B [12]. In this structure, the HC domain wraps around the toxin, giving the protein more compact shape overall. BoNT/E is capable of entering cells much more quickly than BoNT/A [91], and this domain organisation has been proposed to prime the toxin for translocation, resulting in a faster onset of paralysis [12]. However, investigations using various chimeras of BoNT/A1 and BoNT/E1 showed that the speed of translocation is not affected by the binding domain [92].

### 2.6. BoNT/F

In addition to BoNT/F1, there are eight other BoNT/F subtypes (BoNT/F2 to /F9) which differ by up to 30% sequence identity (Table 4). The exact protein receptor for BoNT/F1 has been reported to be glycosylated SV2 [81,100], but this remains to be established conclusively. For example, one study showed that BoNT/F activity decreased when H_C_/A was introduced as a competitor molecule [81], whereas a separate study demonstrated that BoNT/F1 entry in neurones was unaffected by a double SV2A/B knockout in cortical neurones (which have negligible expression of SV2C) [101]. For ganglioside binding, BoNT/F1 requires gangliosides containing an α2,3-linked sialic acid on the terminal galactose (i.e., GT1b or GD1a) [81,100]. The SxWY motif is conserved in BoNT/F, and the crystal structure of the H_C_ domain from BoNT/F1 in complex with GD1a confirmed the existence of a GBS [102].

### 2.7. BoNT/G

Only two protein sequences of BoNT/G are currently known to exist, and they share 99.9% amino acid identity. The protein receptor for BoNT/G is either SytI or SytII, although interestingly the interface diverges from BoNT/B and it has a lower binding affinity [58,106,107]. Only 5 of 14 residues involved in the BoNT/B–SytII interaction are conserved [57,106]. BoNT/G also displays a low affinity for the human SytII receptor due to a human/chimpanzee-specific mutation [61]. The BoNT/B H_C_ domain was successfully engineered to improve human SytII binding, and a similar approach would be worth investigating here [63]. BoNT/G possesses the conserved SxWY motif in its GBS, and binds preferentially to GT1b [108]. In addition to the dual-receptor interactions, BoNT/G also contains a “lipid-binding loop” (residues 1252–1256) similar to that of BoNT/B which can directly interact with the cell membrane to further contribute binding affinity [67,106], and deletion of this loop dramatically decreased neurotoxicity [67].

### 2.8. Mosaic/Chimeric BoNTs

BoNTs composed of domains from different serotypes also exist in nature. The most common of these chimeric toxins are discussed below.

#### 2.8.1. BoNT/CD

BoNT/CD is a mosaic toxin composed of a LC domain and a H_N_ domain that is most similar to BoNT/C and a H_C_ domain that is most similar to BoNT/D. Interestingly the binding domain of BoNT/CD binds synaptosomes more tightly than BoNT/D [79]. This may be due to residues K1118 and K1136 (which differ from the equivalent residues in BoNT/D, E1114 and G1132) since mutation of these lysines results in a dramatic loss in synaptosome binding affinity [109]. Protein residues which may also interact with a ganglioside have also been identified through crystallisation with a sialic acid molecule [110].

#### 2.8.2. BoNT/DC

The BoNT/DC chimera possesses an LC domain and a H_N_ with 96% sequence identity to BoNT/D and a H_C_ domain similar to that of BoNT/C (74% sequence identity) [111,112]. Botulism caused by BoNT/DC is usually found outside of humans in birds and other mammals, but it is also capable of binding human neuronal cells [62,112]. Despite having a binding domain similar to BoNT/C, BoNT/DC binds to either SytI or SytII. This interaction is mediated by hydrophobic residues, and is distinct from that of BoNT/B [113]. The BoNT/DC protein is particularly interesting, as it appears that it may not require complex gangliosides to enter target neurones [114,115]. However, the crystal structure of BoNT/DC in complex with Sialyl-T suggests that BoNT/DC is capable of recognising a single sialic acid, and thus potentially a range of membrane-bound sugars. The structure also reveals the presence of an extended “lipid-binding loop” that is also observed in BoNT/B and BoNT/G [67,114].

#### 2.8.3. BoNT/HA(FA)

BoNT/FA was recently identified in 2014 from a case of infant botulism [116,117]. At the time it was referred to as BoNT/H (and sometimes still as BoNT/HA) due to its non-neutralisable antigenicity [116], and phylogenetic analysis of the *bont* sequences placed the gene in a lineage distinct from other serotypes [117]. The sequence was finally released to the scientific community after a protracted period of data restriction due to supposed safety concerns [118,119,120]. It was determined that the molecule was a mosaic toxin composed of an LC similar to that of BoNT/F5, an H_N_ domain similar to that of BoNT/F1, and an H_C_ domain similar to that of BoNT/A1 [121]. Direct binding of the BoNT/FA HC domain has been confirmed for glycosylated SV2C-LD4 [122], and crystal structures of this binding domain show some slight differences with respect to BoNT/A1 which would be consistent with a decreased affinity towards the protein backbone of SV2 [122,123]. Although no ganglioside-bound structure of BoNT/FA has yet been solved, the structure of the GBS appears to maintain the same fold as that observed for BoNT/A1 [123]. SV2 is likely the protein receptor for BoNT/FA, and direct binding has been confirmed for glycosylated SV2C-LD4 [122]. The BoNT/FA sequence contains mutations with respect to BoNT/A1 which result in decreased affinity towards the protein backbone of SV2, as determined by a pull-down assay against non-glycosylated SV2C, while the equivalent residues involved in glycan binding remain unchanged [122]. The effect of these mutations towards different isoforms of SV2 remains to be seen. The ganglioside-binding site is able to maintain the same fold as BoNT/A1, but no ganglioside-bound structures yet exist, so the exact interactions remain to be determined [123]. In recent assays using cultured rat embryonic spinal cord neurones and rat cortical neurones, BoNT/FA was found to be much more potent than BoNT/A1. However, counterintuitively the toxin was much less potent when assayed using an ex vivo mouse phrenic nerve hemidiaphragm (mPNHD). These results, along with the methods used for each assay, point toward a toxin that may have a slow speed of onset despite a highly active LC [124]. Understanding the interactions of BoNT/FA with its receptors is crucial to both determining what causes intoxication differences and for developing novel therapeutics.

### 2.9. BoNT/X

A strain of *C. botulinum* that was already known to express BoNT/B was recently found to contain the gene for another BoNT molecule that shared low primary sequence identity to other serotypes (<30%)—this was named BoNT/X [125]. It is unknown whether this molecule is capable of causing human botulism, but interestingly, its LC cleaved non-canonical substrates such as VAMP4, VAMP5, and Ykt6 [125]. This suggests that this toxin significantly diverged from other serotypes during its evolution. Despite this, recent structural characterisation of the LC has revealed a core fold common to all BoNTs [126]. Little is known about the BoNT/X H_N_ and H_C_ domains, and considering the novel characteristics of LC, attempts are underway to determine the specific receptor(s) that it targets and how it functions in vivo. The BoNT/X H_C_ does contain an SxWY sequence motif, indicating that it potentially shares similar ganglioside binding characteristics with other BoNTs. Due to its divergence and low sequence similarity to existing BoNTs, structural and functional characterisation could lead to new insights into receptor binding that could be exploited for future therapeutics.

### 2.10. BoNT-Like Proteins

Considering that BoNTs are the deadliest biological agents that exist, it was surprising to find BoNT-like proteins produced by non-*Clostridium* species. The first was found in 2015 and is referred to as “BoNT/Wo”, named after the bacterium that produced it, *Weissella oryzae* SG25 [127,128]. BoNT/Wo cleaves VAMP at a unique location (Trp89–Trp90) [129], but it does not contain any typical BoNT motifs in the receptor-binding domain. This would be consistent with zero reported cases of botulism in humans. Indeed, it has been speculated that BoNT/Wo may instead target SNARE-mediated plant defence systems [128]. More recently, another BoNT-like gene cluster was discovered in the bacterium *Enterococcus faecium*, which is a ubiquitous commensal microorganism commonly found in the gut of mammals. The BoNT-like protein, referred to as BoNT/En or eBoNT/J, possesses many traditional BoNT motifs, including a HExxH zinc-binding motif in the LC and a ganglioside-binding SxWY motif in the HC domain [130,131]. Early studies indicate that rodents do not possesses the receptor(s) for BoNT/En intoxication [130].

## 3. Conclusions

BoNTs are highly specific and potent exotoxins that are being exploited for therapeutic gain. Our knowledge of the molecular aspects of botulinum neurotoxin, such as mechanism of cell targeting and internalisation, is incomplete and mostly limited to only one or two serotypes (i.e., BoNT/A1 and BoNT/B1). We have yet to fully understand the binding mechanism of others and also how subtle amino acid differences may result in differences of intoxication (i.e., between subtypes). From what we know so far, X-ray crystallography has suggested that the mechanism of binding is more complex than was initially thought. It is possible that BoNTs may accommodate heterogeneous glycosylation of their protein receptors and target a variety of gangliosides to ensure successful binding to their target cell type. It is not a trivial task to determine how BoNTs bind to their receptors on neuronal cell membranes, especially when trying to replicate the conditions in vivo. With the recent discovery of new BoNTs and BoNT-like molecules in other bacterial species, this raises questions regarding the evolution of the *bont* gene cluster, their ability to be transferred between species, the potential implications for biosafety, and the need for an agreed-upon consistent naming convention to avoid confusion and ambiguity [132,133]. Fast characterisation and the generation of neutralising antibodies against these novel toxins is required. Despite the potential dangers posed, the knowledge may lead to the generation of new and safer therapeutics. In particular, atomic data of the receptor-binding domains from individual subtypes could be used for structural and functional analyses, providing insights for the design of novel BoNTs [134]. In summary, this review highlights the need for further functional and structural characterisation of different BoNT subtypes to improve our understanding of what determines the toxicological differences and how they may be used in therapeutics.

## Figures and Tables

**Table 1:** BoNT/A subtype primary sequence identities. Percentage identities are given for full-length and H_C_ domain sequence alignments of each BoNT/A subtype. For the H_C_ alignments, sequences aligned to BoNT/A1 residues 870–1296 were used. Uniprot accession codes for BoNT/A1 to /A8 are A5HZZ9 [45,46,47], Q45894 [48], Q3LRX9 [49], Q3LRX8 [49], C7BEA8 [50], C9WWY7 [51], K4LN57 [52], and A0A0A7PDB7 [53], respectively.

	A1	A2	A3	A4	A5	A6	A7	A8	
**A1**	–	87.29	86.82	91.55	93.90	90.61	91.78	87.79	**H_C_ identity**
**A2**	90.04	–	98.83	88.47	89.65	90.12	90.35	93.43	
**A3**	84.66	93.19	–	88.24	88.94	89.65	89.88	92.72	
**A4**	89.19	88.12	84.29	–	86.85	85.92	85.92	90.14	
**A5**	97.15	90.50	85.21	87.34	–	93.43	92.72	89.91	
**A6**	95.68	91.74	86.29	87.72	95.91	–	91.08	87.32	
**A7**	93.75	89.81	84.90	86.64	94.37	92.98	–	89.67	
**A8**	93.36	93.44	87.69	88.81	93.60	93.06	91.36	–	
	**Full-length identity**								

**Table 2:** BoNT/B subtype primary sequence identities. Percentage identities are given for full-length and H_C_ domain sequence alignments of each BoNT/B subtype. For the H_C_ alignments, sequences aligned to BoNT/B1 residues 862–1291 were used. UniprotKB accession codes for BoNT/B1 to /B8 are P10844 [68], A2I2R7 [69], A2I2S2 [69], A2I2R6 [69], A2I2R9 [70], A8R089 [71], H9CNK9 [72], I6Z8G9 [54], respectively.

	B1	B2	B3	B4	B5	B6	B7	B8	
**B1**	–	91.86	93.02	89.53	95.35	93.72	90.23	92.33	**H_C_ identity**
**B2**	95.66	–	96.05	91.16	91.40	96.74	93.02	93.02	
**B3**	95.97	98.45	–	90.47	92.79	96.28	92.79	92.79	
**B4**	93.41	94.19	94.03	–	88.60	89.77	90.70	89.07	
**B5**	96.13	95.20	95.51	92.87	–	93.26	90.23	91.86	
**B6**	96.20	98.45	98.22	93.49	95.41	–	91.86	92.56	
**B7**	94.81	95.89	95.74	93.88	94.19	95.20	–	90.70	
**B8**	95.51	95.97	95.82	93.34	94.50	95.66	94.58	–	
	**Full-length identity**								

**Table 3:** BoNT/E primary sequence identities. Percentage identities are given for full-length and H_C_ domain sequence alignments of each BoNT/E subtype. For the H_C_ alignments, sequences aligned to BoNT/E1 residues 848–1252 were used. Uniprot accession codes for BoNT/E1 to /E12 are K7S1V3 [93], A2I2S6 [93], A2I2S5 [93], C4IHM1 [94], Q9K395 [95], A8Y878 [96], G8I2N7 [93], G8I2N8 [93,97], K7S9Y2 [93], A0A076JVL9 [98], A0A076K0B0 [98], W8FNB6 [99], respectively.

	E1	E2	E3	E4	E5	E6	E7	E8	E9	E10	E11	E12	
**E1**	–	97.53	100.0	97.78	92.82	97.28	100.0	92.28	83.17	94.57	93.58	87.87	**H_C_ identity**
**E2**	99.20	–	97.53	97.28	91.34	96.79	97.53	99.75	84.16	95.80	95.31	88.61	
**E3**	98.16	97.36	–	97.78	92.82	97.28	100.0	92.28	83.17	94.57	93.58	87.87	
**E4**	97.28	97.12	95.69	–	90.84	99.51	97.78	97.53	82.67	94.07	93.09	86.88	
**E5**	96.88	96.40	95.20	95.04	–	90.84	92.82	91.09	83.91	88.86	89.60	90.84	
**E6**	96.96	96.81	95.93	96.96	94.88	–	97.28	97.04	82.67	93.83	93.09	86.88	
**E7**	97.92	97.12	97.36	96.25	94.88	96.41	–	97.28	83.17	94.57	93.58	87.87	
**E8**	96.25	97.04	95.69	96.17	94.16	96.81	98.32	–	83.91	96.05	95.56	88.37	
**E9**	89.05	89.37	88.73	90.01	89.45	88.25	89.21	89.45	–	83.66	85.15	88.37	
**E10**	95.37	95.77	94.81	94.97	93.53	95.69	96.88	97.84	89.45	–	97.04	87.38	
**E11**	93.29	93.85	92.57	92.73	92.01	93.13	93.45	94.41	89.05	95.61	–	87.62	
**E12**	92.97	93.21	92.65	92.65	93.61	91.21	92.57	92.09	91.45	92.01	91.13	–	
	**Full-length identity**												

**Table 4:** BoNT/F subtype primary sequence identities. Percentage identities are given for full-length and H_C_ domain sequence alignments of each BoNT/F subtype. For the H_C_ alignments, sequences aligned to BoNT/F1 residues 866–1278 were used. Uniprot accession codes for BoNT/F1 to /F9 are A7GBG3 [103], D2KHQ7 [103], D2KHR6 [103], D2KHQ8 [103], D2KHQ9 [103], D2KHS4 [103], D2KHS9 [103], KEJ01913 ${}^{*}$ [104], A0A1P8YWK9 [105], respectively. ${}^{*}$ GenBank accession code given where UniProtKB code not available.

	F1	F2	F3	F4	F5	F6	F7	F8	F9	
**F1**	–	82.97	84.63	89.29	83.94	82.51	79.56	98.05	84.71	**H_C_ identity**
**F2**	83.71	–	96.37	81.51	92.03	93.89	72.02	83.70	90.34	
**F3**	84.25	97.19	–	82.44	93.46	93.64	73.17	84.39	92.49	
**F4**	92.33	83.71	84.09	–	82.00	81.03	76.40	88.56	83.45	
**F5**	70.31	74.37	74.35	69.84	–	90.22	73.48	84.18	92.03	
**F6**	88.05	90.20	90.04	87.42	74.11	–	72.17	82.27	88.75	
**F7**	74.43	69.53	69.91	72.77	64.45	70.84	–	79.32	73.48	
**F8**	96.24	83.71	84.17	93.19	69.84	87.81	73.01	–	84.67	
**F9**	84.27	89.92	81.63	84.03	73.75	87.37	69.85	84.18	–	
	**Full-length identity**									

## References

[1] Hatheway C.L. (1990). Toxigenic clostridia. Clin. Microbiol. Rev..

[2] Collins M.D., East A.K. (1998). Phylogeny and taxonomy of the food-borne pathogen *Clostridium botulinum* and its neurotoxins. J. Appl. Microbiol..

[3] Carter A.T., Peck M.W. (2015). Genomes, neurotoxins and biology of *Clostridium botulinum* Group I and Group II. Res. Microbiol..

[4] DasGupta B.R., Sugiyama H. (1972). Role of a protease in natural activation of *Clostridium botulinum* neurotoxin. Infect. Immun..

[5] Prabakaran S., Tepp W., DasGupta B.R. (2001). Botulinum neurotoxin types B and E: purification, limited proteolysis by endoproteinase Glu-C and pepsin, and comparison of their identified cleaved sites relative to the three-dimensional structure of type A neurotoxin. Toxicon.

[6] Dekleva M.L., DasGupta B.R. (1989). Nicking of single chain *Clostridium botulinum* type A neurotoxin by an endogenous protease. Biochem. Biophys. Res. Commun..

[7] Dekleva M.L., DasGupta B.R. (1990). Purification and characterization of a protease from *Clostridium botulinum* type A that nicks single-chain type A botulinum neurotoxin into the di-chain form. J. Bacteriol..

[8] Fischer A., Montal M. (2007). Crucial role of the disulfide bridge between botulinum neurotoxin light and heavy chains in protease translocation across membranes. J. Biol. Chem..

[9] Pirazzini M., Rossetto O., Bolognese P., Shone C.C., Montecucco C. (2011). Double anchorage to the membrane and intact inter-chain disulfide bond are required for the low pH induced entry of tetanus and botulinum neurotoxins into neurons. Cell. Microbiol..

[10] Lacy D.B., Tepp W., Cohen A.C., DasGupta B.R., Stevens R.C. (1998). Crystal structure of botulinum neurotoxin type A and implications for toxicity. Nat. Struct. Biol..

[11] Swaminathan S., Eswaramoorthy S. (2000). Structural analysis of the catalytic and binding sites of *Clostridium botulinum* neurotoxin B. Nat. Struct. Biol..

[12] Kumaran D., Eswaramoorthy S., Furey W., Navaza J., Sax M., Swaminathan S. (2009). Domain organization in *Clostridium botulinum* neurotoxin type E is unique: Its implication in faster translocation. J. Mol. Biol..

[13] Montecucco C. (1986). How do tetanus and botulinum toxins bind to neuronal membranes?. Trends Biochem. Sci..

[14] Brunger A.T., Rummel A. (2009). Receptor and substrate interactions of clostridial neurotoxins. Toxicon.

[15] Morgans C.W., Kensel-Hammes P., Hurley J.B., Burton K., Idzerda R., McKnight G.S., Bajjalieh S.M. (2009). Loss of the synaptic vesicle protein SV2B results in reduced neurotransmission and altered synaptic vesicle protein expression in the retina. PLoS ONE.

[16] Chapman E.R. (2002). Synaptotagmin: A Ca^2+^ sensor that triggers exocytosis?. Nat. Rev. Mol. Cell Biol..

[17] Tang J., Maximov A., Shin O., Dai H., Rizo J., Sudhof T.C. (2006). A complexin/synaptotagmin 1 switch controls fast synaptic vesicle exocytosis. Cell.

[18] Chen C., Arai I., Satterfield R., Young S.M., Jonas P. (2017). Synaptotagmin 2 Is the Fast Ca^2+^ sensor at a central inhibitory synapse. Cell Rep..

[19] Südhof T.C. (2002). Synaptotagmins: Why so many?. J. Biol. Chem..

[20] Koriazova L.K., Montal M. (2003). Translocation of botulinum neurotoxin light chain protease through the heavy chain channel. Nat. Struct. Biol..

[21] Pirazzini M., Azarnia T.D., Leka O., Zanetti G., Rossetto O., Montecucco C. (2016). On the translocation of botulinum and tetanus neurotoxins across the membrane of acidic intracellular compartments. Biochim. Biophys. Acta.

[22] Fischer A., Sambashivan S., Brunger A.T., Montal M. (2012). Beltless translocation domain of botulinum neurotoxin A embodies a minimum ion-conductive channel. J. Biol. Chem..

[23] Pirazzini M., Azarnia T.D., Zanetti G., Megighian A., Scorzeto M., Fillo S., Shone C.C., Binz T., Rossetto O., Lista F. (2014). Thioredoxin and its reductase are present on synaptic vesicles, and their inhibition prevents the paralysis induced by botulinum neurotoxins. Cell Rep..

[24] Zanetti G., Pirazzini M., Binz T., Shone C.C., Fillo S., Lista F., Rossetto O., Montecucco C. (2015). Inhibition of botulinum neurotoxins interchain disulfide bond reduction prevents the peripheral neuroparalysis of botulism. Biochem. Pharmacol..

[25] Johnson E.A. (1999). Clostridial toxins as therapeutic agents: Benefits of nature’s most toxic proteins. Annu. Rev. Microbiol..

[26] Montecucco C., Molgó J. (2005). Botulinal neurotoxins: Revival of an old killer. Curr. Opin. Pharmacol..

[27] Foster K.A. (2014). Molecular Aspects of Botulinum Neurotoxin. Current Topics in Neurotoxicity.

[28] Rummel A., Mahrhold S., Bigalke H., Binz T. (2004). The HCC-domain of botulinum neurotoxins A and B exhibits a singular ganglioside binding site displaying serotype specific carbohydrate interaction. Mol. Microbiol..

[29] Yowler B.C., Kensinger R.D., Schengrund C.L. (2002). Botulinum neurotoxin A activity is dependent upon the presence of specific gangliosides in neuroblastoma cells expressing synaptotagmin I. J. Biol. Chem..

[30] Hamark C., Berntsson R.P., Masuyer G., Henriksson L.M., Gustafsson R., Stenmark P., Widmalm G. (2017). Glycans confer specificity to the recognition of ganglioside receptors by botulinum neurotoxin A. J. Am. Chem. Soc..

[31] Stenmark P., Dupuy J., Imamura A., Kiso M., Stevens R.C. (2008). Crystal structure of botulinum neurotoxin type A in complex with the cell surface co-receptor GT1b-insight into the toxin-neuron interaction. PLoS Pathog..

[32] Dong M., Yeh F., Tepp W.H., Dean C., Johnson E.A., Janz R., Chapman E.R. (2006). SV2 is the protein receptor for botulinum neurotoxin A. Science.

[33] Mahrhold S., Rummel A., Bigalke H., Davletov B., Binz T. (2006). The synaptic vesicle protein 2C mediates the uptake of botulinum neurotoxin A into phrenic nerves. FEBS Lett..

[34] Benoit R.M., Frey D., Hilbert M., Kevenaar J.T., Wieser M.M., Stirnimann C.U., McMillan D., Ceska T., Lebon F., Jaussi R. (2014). Structural basis for recognition of synaptic vesicle protein 2C by botulinum neurotoxin A. Nature.

[35] Mahrhold S., Bergström T., Stern D., Dorner B.G., Åstot C., Rummel A. (2016). Only the complex N559-glycan in the synaptic vesicle glycoprotein 2C mediates high affinity binding to botulinum neurotoxin serotype A1. Biochem. J..

[36] Dong M., Liu H., Tepp W.H., Johnson E.A., Janz R., Chapman E.R. (2008). Glycosylated SV2A and SV2B mediate the entry of botulinum neurotoxin E into neurons. Mol. Biol. Cell.

[37] Yao G., Zhang S., Mahrhold S., Lam K.H., Stern D., Bagramyan K., Perry K., Kalkum M., Rummel A., Dong M. (2016). N-linked glycosylation of SV2 is required for binding and uptake of botulinum neurotoxin A. Nat. Struct. Mol. Biol..

[38] Benoit R.M., Schärer M.A., Wieser M.M., Li X., Frey D., Kammerer R.A. (2017). Crystal structure of the BoNT/A2 receptor-binding domain in complex with the luminal domain of its neuronal receptor SV2C. Sci. Rep..

[39] Gustafsson R., Zhang S., Masuyer G., Dong M., Stenmark P. (2018). Crystal structure of botulinum neurotoxin A2 in complex with the human protein receptor SV2C reveals plasticity in receptor binding. Toxins (Basel).

[40] Kroken A., Blum F., Zuverink M., Barbieri J. (2017). Entry of botulinum neurotoxin subtypes A1 and A2 into neurons. Infect. Immun..

[41] Davies J.R., Rees J., Liu S.M., Acharya K.R. (2018). High resolution crystal structures of *Clostridium botulinum* neurotoxin A3 and A4 binding domains. J. Struct. Biol..

[42] Pier C.L., Chen C., Tepp W.H., Lin G., Janda K.D., Barbieri J.T., Pellett S., Johnson E.A. (2011). Botulinum neurotoxin subtype A2 enters neuronal cells faster than subtype A1. FEBS Lett..

[43] Torii Y., Kiyota N., Sugimoto N., Mori Y., Goto Y., Harakawa T., Nakahira S., Kaji R., Kozaki S., Ginnaga A. (2011). Comparison of effects of botulinum toxin subtype A1 and A2 using twitch tension assay and rat grip strength test. Toxicon.

[44] Pellett S., Tepp W.H., Whitemarsh R.C., Bradshaw M., Johnson E.A. (2015). In vivo onset and duration of action varies for botulinum neurotoxin A subtypes 1–5. Toxicon.

[45] Betley M.J., Somers E., DasGupta B.R. (1989). Characterization of botulinum type a neurotoxin gene: Delineation of the N-terminal encoding region. Biochem. Biophys. Res. Commun..

[46] Gimenez J.A., DasGupta B.R. (1993). Botulinum type A neurotoxin digested with pepsin yields 132, 97, 72, 45, 42, and 18 kD fragments. J. Prot. Chem..

[47] Smith T.J., Hill K.K., Foley B.T., Detter J.C., Munk A.C., Bruce D.C., Doggett N.A., Smith L.A., Marks J.D., Xie G. (2007). Analysis of the neurotoxin complex genes in *Clostridium botulinum* A1–A4 and B1 strains: BoNT/A3, /Ba4 and /B1 clusters are located within plasmids. PLoS ONE.

[48] Dover N., Barash J.R., Hill K.K., Davenport K.W., Teshima H., Xie G., Arnon S.S. (2013). *Clostridium botulinum* strain Af84 contains three neurotoxin gene clusters: Bont/A2, bont/F4 and bont/F5. PLoS ONE.

[49] Jacobson M.J., Lin G., Raphael B., Andreadis J., Johnson E.A. (2008). Analysis of neurotoxin cluster genes in *Clostridium botulinum* strains producing botulinum neurotoxin serotype A subtypes. Appl. Environ. Microbiol..

[50] Dover N., Barash J.R., Arnon S.S. (2009). Novel *Clostridium botulinum* toxin gene arrangement with subtype A5 and partial subtype B3 botulinum neurotoxin genes. J. Clin. Microbiol..

[51] Luquez C., Raphael B.H., Maslanka S.E. (2009). Neurotoxin gene clusters in *Clostridium botulinum* type Ab strains. Appl. Environ. Microbiol..

[52] Mazuet C., Ezan E., Volland H., Popoff M.R., Becher F. (2012). Toxin detection in patients’ sera by mass spectrometry during two outbreaks of type A botulism in France. J. Clin. Microbiol..

[53] Kull S., Schulz K.M., Weisemann J., Kirchner S., Schreiber T., Bollenbach A., Dabrowski P.W., Nitsche A., Kalb S.R., Dorner M.B. (2015). Isolation and functional characterization of the novel *Clostridium botulinum* neurotoxin A8 subtype. PLoS ONE.

[54] Wangroongsarb P., Kohda T., Jittaprasartsin C., Suthivarakom K., Kamthalang T., Umeda K., Sawanpanyalert P., Kozaki S., Ikuta K. (2014). Molecular characterization of *Clostridium botulinum* isolates from foodborne outbreaks in Thailand, 2010. PLoS ONE.

[55] Nishiki T., Tokuyama Y., Kamata Y., Nemoto Y., Yoshida A., Sekiguchi M., Takahashi M., Kozaki S. (1996). Binding of botulinum type B neurotoxin to Chinese hamster ovary cells transfected with rat synaptotagmin II cDNA. Neurosci. Lett..

[56] Dong M., Richards D.A., Goodnough M.C., Tepp W.H., Johnson E.A., Chapman E.R. (2003). Synaptotagmins I and II mediate entry of botulinum neurotoxin B into cells. J. Cell Biol..

[57] Rummel A., Eichner T., Weil T., Karnath T., Gutcaits A., Mahrhold S., Sandhoff K., Proia R.L., Acharya K.R., Bigalke H. (2007). Identification of the protein receptor binding site of botulinum neurotoxins B and G proves the double-receptor concept. Proc. Natl. Acad. Sci. USA.

[58] Dong M., Tepp W.H., Liu H., Johnson E.A., Chapman E.R. (2007). Mechanism of botulinum neurotoxin B and G entry into hippocampal neurons. J. Cell Biol..

[59] Chai Q., Arndt J.W., Dong M., Tepp W.H., Johnson E.A., Chapman E.R., Stevens R.C. (2006). Structural basis of cell surface receptor recognition by botulinum neurotoxin B. Nature.

[60] Jin R., Rummel A., Binz T., Brunger A. (2006). Botulinum neurotoxin B recognizes its protein receptor with high affinity and specificity. Nature.

[61] Strotmeier J., Willjes G., Binz T., Rummel A. (2012). Human synaptotagmin-II is not a high affinity receptor for botulinum neurotoxin B and G: increased therapeutic dosage and immunogenicity. FEBS Lett..

[62] Peng L., Berntsson R.P., Tepp W.H., Pitkin R.M., Johnson E.A., Stenmark P., Dong M. (2012). Botulinum neurotoxin D-C uses synaptotagmin I and II as receptors, and human synaptotagmin II is not an effective receptor for type B, D-C and G toxins. J. Cell Sci..

[63] Tao L., Peng L., Berntsson R.P., Liu S.M., Park S., Yu F., Boone C., Palan S., Beard M., Chabrier P. (2017). Engineered botulinum neurotoxin B with improved efficacy for targeting human receptors. Nat. Commun..

[64] Kohda T., Ihara H., Seto Y., Tsutsuki H., Mukamoto M., Kozaki S. (2007). Differential contribution of the residues in C-terminal half of the heavy chain of botulinum neurotoxin type B to its binding to the ganglioside GT1b and the synaptotagmin 2/GT1b complex. Microb. Pathog..

[65] Berntsson R.P., Peng L., Dong M., Stenmark P. (2013). Structure of dual receptor binding to botulinum neurotoxin B. Nat. Commun..

[66] Atassi M.Z., Taruishi M., Naqvi M., Steward L.E., Aoki K.R. (2014). Synaptotagmin II and gangliosides bind independently with botulinum neurotoxin B but each restrains the other. Protein J..

[67] Stern D., Weisemann J., Le Blanc A., von Berg L., Mahrhold S., Piesker J., Laue M., Luppa P.B., Dorner M.B., Dorner B.G. (2018). A lipid-binding loop of botulinum neurotoxin serotypes B, DC and G is an essential feature to confer their exquisite potency. PLoS Pathog..

[68] Whelan S.M., Elmore M.J., Bodsworth N.J., Brehm J.K., Atkinson T., Minton N.P. (1992). Molecular cloning of the *Clostridium botulinum* structural gene encoding the type B neurotoxin and determination of its entire nucleotide sequence. Appl. Environ. Microbiol..

[69] Hill K.K., Smith T.J., Helma C.H., Ticknor L.O., Foley B.T., Svensson R.T., Brown J.L., Johnson E.A., Smith L.A., Okinaka R.T. (2007). Genetic diversity among Botulinum Neurotoxin-producing clostridial strains. J. Bacteriol..

[70] Kenri T., Sekizuka T., Yamamoto A., Iwaki M., Komiya T., Hatakeyama T., Nakajima H., Takahashi M., Kuroda M., Shibayama K. (2014). Genetic characterization and comparison of *Clostridium botulinum* isolates from botulism cases in Japan between 2006 and 2011. Appl. Environ. Microbiol..

[71] Kohda T., Nakamura K., Hosomi K., Torii Y., Kozaki S., Mukamoto M. (2017). Characterization of the functional activity of botulinum neurotoxin subtype B6. Microbiol. Immunol..

[72] Kalb S.R., Baudys J., Rees J.C., Smith T.J., Smith L.A., Helma C.H., Hill K., Kull S., Kirchner S., Dorner M.B. (2012). De novo subtype and strain identification of botulinum neurotoxin type B through toxin proteomics. Anal. Bioanal. Chem..

[73] Lindström M., Nevas M., Kurki J., Sauna-aho R., Latvala-Kiesilä A., Pölönen I., Korkeala H. (2004). Type C botulism due to toxic feed affecting 52,000 farmed foxes and minks in Finland. J. Clin. Microbiol..

[74] Hauser D., Eklund M.W., Kurazono H., Binz T., Niemann H., Gill D.M., Boquet P., Popoff M. (1990). Nucleotide sequence of *Clostridium botulinum* C1 neurotoxin. Nucleic Acids Res..

[75] Takeda M., Tsukamoto K., Kohda T., Matsui M., Mukamoto M., Kozaki S. (2005). Characterization of the neurotoxin produced by isolates associated with avian botulism. Avian Dis..

[76] Stiles B.G., Pradhan K., Fleming J.M., Samy R.P., Barth H., Popoff M.R. (2014). Clostridium and bacillus binary enterotoxins: Bad for the bowels, and eukaryotic being. Toxins (Basel).

[77] Chellapandi P., Prisilla A. (2017). Structure, function and evolution of *Clostridium botulinum* C2 and C3 toxins: Insight to poultry and veterinary vaccines. Curr. Protein Pept. Sci..

[78] Evans H.R., Holloway D.E., Sutton J.M., Ayriss J., Shone C.C., Acharya K.R. (2004). C3 exoenzyme from *Clostridium botulinum*: Structure of a tetragonal crystal form and a reassessment of NAD-induced flexure. Acta Crystallogr. D Biol. Crystallogr..

[79] Tsukamoto K., Kohda T., Mukamoto M., Takeuchi K., Ihara H., Saito M., Kozaki S. (2005). Binding of *Clostridium botulinum* type C and D neurotoxins to ganglioside and phospholipid. Novel insights into the receptor for clostridial neurotoxins. J. Biol. Chem..

[80] Kroken A.R., Karalewitz A.P., Fu Z., Baldwin M.R., Kim J.J., Barbieri J.T. (2011). Unique ganglioside binding by botulinum neurotoxins C and D-SA. FEBS J..

[81] Rummel A., Häfner K., Mahrhold S., Darashchonak N., Holt M., Jahn R., Beermann S., Karnath T., Bigalke H., Binz T. (2009). Botulinum neurotoxins C, E and F bind gangliosides via a conserved binding site prior to stimulation-dependent uptake with botulinum neurotoxin F utilising the three isoforms of SV2 as second receptor. J. Neurochem..

[82] Strotmeier J., Gu S., Jutzi S., Mahrhold S., Zhou J., Pich A., Eichner T., Bigalke H., Rummel A., Jin R. (2011). The biological activity of botulinum neurotoxin type C is dependent upon novel types of ganglioside binding sites. Mol. Microbiol..

[83] Karalewitz A.P., Fu Z., Baldwin M.R., Kim J.J., Barbieri J.T. (2012). Botulinum neurotoxin serotype C associates with dual ganglioside receptors to facilitate cell entry. J. Biol. Chem..

[84] Peng L., Tepp W.H., Johnson E.A., Dong M. (2011). Botulinum neurotoxin D uses synaptic vesicle protein SV2 and gangliosides as receptors. PLoS Pathog..

[85] Strotmeier J., Lee K., Völker A.K., Mahrhold S., Zong Y., Zeiser J., Zhou J., Pich A., Bigalke H., Binz T. (2010). Botulinum neurotoxin serotype D attacks neurons via two carbohydrate-binding sites in a ganglioside-dependent manner. Biochem. J..

[86] Zhang Y., Buchko G.W., Qin L., Robinson H., Varnum S.M. (2010). Structural analysis of the receptor binding domain of botulinum neurotoxin serotype D. Biochem. Biophys. Res. Commun..

[87] Kroken A.R., Karalewitz A.P., Fu Z., Kim J.J., Barbieri J.T. (2011). Novel ganglioside-mediated entry of botulinum neurotoxin serotype D into neurons. J. Biol. Chem..

[88] Stefan M., Jasmin S., Consuelo G., Jianlong L., James D.M., Andreas R., Thomas B., Mahrhold S., Strotmeier J., Garcia-Rodriguez C. (2013). Identification of the SV2 protein receptor-binding site of botulinum neurotoxin type E. Biochem. J..

[89] Kamata Y., Kozaki S., Sakaguchi G., Iwamori M., Nagai Y. (1986). Evidence for direct binding of *Clostridium botulinum* type E derivative toxin and its fragments to gangliosides and free fatty acids. Biochem. Biophys. Res. Commun..

[90] Sun S., Tepp W.H., Johnson E.A., Chapman E.R. (2012). Botulinum neurotoxins B and E translocate at different rates and exhibit divergent responses to GT1b and low pH. Biochemistry.

[91] Keller J.E., Cai F., Neale E.A. (2004). Uptake of botulinum neurotoxin into cultured neurons. Biochemistry.

[92] Wang J., Meng J., Lawrence G.W., Zurawski T.H., Sasse A., Bodeker M.O., Gilmore M.A., Fernández-Salas E., Francis J., Steward L.E. (2008). Novel chimeras of botulinum neurotoxins A and E unveil contributions from the binding, translocation, and protease domains to their functional characteristics. J. Biol. Chem..

[93] Raphael B.H., Lautenschlager M., Kalb S.R., de Jong L.I., Frace M., Lúquez C., Barr J.R., Fernández R.A., Maslanka S.E. (2012). Analysis of a unique *Clostridium botulinum* strain from the Southern hemisphere producing a novel type E botulinum neurotoxin subtype. BMC Microbiol..

[94] Dykes J.K., Lúquez C., Raphael B.H., McCroskey L., Maslanka S.E. (2015). Laboratory investigation of the first case of botulism caused by *Clostridium butyricum* type E toxin in the United States. J. Clin. Microbiol..

[95] Wang X., Maegawa T., Karasawa T., Kozaki S., Tsukamoto K., Gyobu Y., Yamakawa K., Oguma K., Sakaguchi Y., Nakamura S. (2000). Genetic analysis of type E botulinum toxin-producing *Clostridium butyricum* strains. Appl. Environ. Microbiol..

[96] Chen Y., Korkeala H., Aarnikunnas J., Lindström M. (2007). Sequencing the botulinum neurotoxin gene and related genes in *Clostridium botulinum* type E strains reveals orfx3 and a novel type E neurotoxin subtype. J. Bacteriol..

[97] Macdonald T.E., Helma C.H., Shou Y., Valdez Y.E., Ticknor L.O., Foley B.T., Davis S.W., Hannett G.E., Kelly-Cirino C.D., Barash J.R. (2011). Analysis of *Clostridium botulinum* serotype E strains by using multilocus sequence typing, amplified fragment length polymorphism, variable-number tandem-repeat analysis, and botulinum neurotoxin gene sequencing. Appl. Environ. Microbiol..

[98] Weedmark K.A., Lambert D.L., Mabon P., Hayden K.L., Urfano C.J., Leclair D., Van Domselaar G., Austin J.W., Corbett C.R. (2014). Two novel toxin variants revealed by whole-genome sequencing of 175 *Clostridium botulinum* type E strains. Appl. Environ. Microbiol..

[99] Mazuet C., Sautereau J., Legeay C., Bouchier C., Bouvet P., Popoff M.R. (2015). An atypical outbreak of food-borne botulism due to *Clostridium botulinum* types B and E from ham. J. Clin. Microbiol..

[100] Fu Z., Chen C., Barbieri J.T., Kim J.P., Baldwin M.R. (2009). Glycosylated SV2 and gangliosides as dual receptors for botulinum neurotoxin serotype F. Biochemistry.

[101] Yeh F.L., Dong M., Yao J., Tepp W.H., Lin G., Johnson E.A., Chapman E.R. (2010). SV2 mediates entry of tetanus neurotoxin into central neurons. PLoS Pathog..

[102] Benson M.A., Fu Z., Kim J.J., Baldwin M.R. (2011). Unique ganglioside recognition strategies for clostridial neurotoxins. J. Biol. Chem..

[103] Raphael B.H., Choudoir M.J., Lúquez C., Fernández R., Maslanka S.E. (2010). Sequence diversity of genes encoding botulinum neurotoxin type F. Appl. Environ. Microbiol..

[104] Giordani F., Fillo S., Anselmo A., Palozzi A.M., Fortunato A., Gentile B., Azarnia T.D., Ciammaruconi A., Spagnolo F., Pittiglio V. (2015). Genomic characterization of Italian *Clostridium botulinum* group I strains. Infect. Genet. Evol..

[105] Sikorra S., Skiba M., Dorner M.B., Weisemann J., Weil M., Valdezate S., Davletov B., Rummel A., Dorner B., Binz T. (2018). Botulinum neurotoxin F subtypes cleaving the VAMP-2 Q-K peptide bond exhibit unique catalytic properties and substrate specificities. Toxins (Basel).

[106] Stenmark P., Dong M., Dupuy J., Chapman E.R., Stevens R.C. (2010). Crystal structure of the botulinum neurotoxin type G binding domain: Insight into cell surface binding. J. Mol. Biol..

[107] Willjes G., Mahrhold S., Strotmeier J., Eichner T., Rummel A., Binz T. (2013). Botulinum neurotoxin G binds synaptotagmin-II in a mode similar to that of serotype B: Tyrosine 1186 and lysine 1191 cause its lower affinity. Biochemistry.

[108] Schmitt J., Karalewitz A., Benefield D.A., Mushrush D.J., Pruitt R.N., Spiller B.W., Barbieri J.T., Lacy D.B. (2010). Structural analysis of botulinum neurotoxin type G receptor binding. Biochemistry.

[109] Zhang Y., Buchko G.W., Qin L., Robinson H., Varnum S.M. (2011). Crystal structure of the receptor binding domain of the botulinum C-D mosaic neurotoxin reveals potential roles of lysines 1118 and 1136 in membrane interactions. Biochem. Biophys. Res. Commun..

[110] Zhang Y., Gardberg A.S., Edwards T.E., Sankaran B., Robinson H., Varnum S.M., Buchko G.W. (2013). Structural insights into the functional role of the Hcn sub-domain of the receptor-binding domain of the botulinum neurotoxin mosaic serotype C/D. Biochimie.

[111] Moriishi K., Koura M., Abe N., Fujii N., Fujinaga Y., Inoue K., Ogumad K. (1996). Mosaic structures of neurotoxins produced from *Clostridium botulinum* types C and D organisms. Biochim. Biophys. Acta.

[112] Nakamura K., Kohda T., Umeda K., Yamamoto H., Mukamoto M., Kozaki S. (2010). Characterization of the D/C mosaic neurotoxin produced by *Clostridium botulinum* associated with bovine botulism in Japan. Vet. Microbiol..

[113] Berntsson R.P., Peng L., Svensson L.M., Dong M., Stenmark P. (2013). Crystal structures of botulinum neurotoxin DC in complex with its protein receptors synaptotagmin I and II. Structure.

[114] Zhang S., Berntsson R., Tepp W., Tao L., Johnson E., Stenmark P., Dong M. (2017). Structural basis for the unique ganglioside and cell membrane recognition mechanism of botulinum neurotoxin DC. Nat. Commun..

[115] Karalewitz A.P., Kroken A.R., Fu Z., Baldwin M.R., Kim J.J., Barbieri J.T. (2010). Identification of a unique ganglioside binding loop within botulinum neurotoxins C and D-SA. Biochemistry.

[116] Barash J.R., Arnon S.S. (2014). A novel strain of *Clostridium botulinum* that produces type B and type H botulinum toxins. J. Infect. Dis..

[117] Dover N., Barash J.R., Hill K.K., Xie G., Arnon S.S. (2014). Molecular characterization of a novel botulinum neurotoxin type H gene. J. Infect. Dis..

[118] Hooper D.C., Hirsch M.S. (2014). Novel *Clostridium botulinum* Toxin and Dual Use Research of Concern Issues. J. Infect. Dis..

[119] Relman D.A. (2014). “Inconvenient Truths” in the pursuit of scientific knowledge and public health. J. Infect. Dis..

[120] Johnson E.A. (2014). Validity of botulinum neurotoxin serotype H. J. Infect. Dis..

[121] Gonzalez-Escalona N., Thirunavukkarasu N., Singh A., Toro M., Brown E., Zink D., Rummel A., Sharma S.K. (2014). Draft genome sequence of bivalent *Clostridium botulinum* strain IBCA10-7060, encoding botulinum neurotoxin B and a new FA mosaic type. Genome Announc..

[122] Yao G., Lam K.H., Perry K., Weisemann J., Rummel A., Jin R. (2017). Crystal structure of the receptor-binding domain of botulinum neurotoxin type HA, also known as type FA or H. Toxins (Basel).

[123] Davies J.R., Hackett G.S., Liu S.M., Acharya K.R. (2018). High resolution crystal structures of the receptor-binding domain of *Clostridium botulinum* neurotoxin serotypes A and FA. PeerJ.

[124] Hackett G., Moore K., Burgin D., Hornby F., Gray B., Elliott M., Mir I., Beard M. (2018). Purification and characterization of recombinant botulinum neurotoxin serotype FA, also known as serotype H. Toxins (Basel).

[125] Zhang S., Masuyer G., Zhang J., Shen Y., Lundin D., Henriksson L., Miyashita S.I., Martínez-Carranza M., Dong M., Stenmark P. (2017). Identification and characterization of a novel botulinum neurotoxin. Nat. Commun..

[126] Masuyer G., Zhang S., Barkho S., Shen Y., Henriksson L., Košenina S., Dong M., Stenmark P. (2018). Structural characterisation of the catalytic domain of botulinum neurotoxin X - high activity and unique substrate specificity. Sci. Rep..

[127] Tanizawa Y., Fujisawa T., Mochizuki T., Kaminuma E., Suzuki Y., Nakamura Y., Tohno M. (2014). Draft genome sequence of *Weissella oryzae* SG25T, isolated from fermented rice grains. Genome Announc..

[128] Mansfield M.J., Adams J.B., Doxey A.C. (2015). Botulinum neurotoxin homologs in non-Clostridium species. FEBS Lett..

[129] Zornetta I., Azarnia Tehran D., Arrigoni G., Anniballi F., Bano L., Leka O., Zanotti G., Binz T., Montecucco C. (2016). The first non *Clostridial botulinum*-like toxin cleaves VAMP within the juxtamembrane domain. Sci. Rep..

[130] Zhang S., Lebreton F., Mansfield M.J., Miyashita S.I., Zhang J., Schwartzman J.A., Tao L., Masuyer G., Martínez-Carranza M., Stenmark P. (2018). Identification of a botulinum neurotoxin-like toxin in a commensal strain of *Enterococcus faecium*. Cell Host Microbe.

[131] Brunt J., Carter A.T., Stringer S.C., Peck M.W. (2018). Identification of a novel botulinum neurotoxin gene cluster in Enterococcus. FEBS Lett..

[132] Mansfield M., Doxey A. (2018). Genomic insights into the evolution and ecology of botulinum neurotoxins. Pathog. Dis..

[133] Williamson C.H.D., Vazquez A.J., Hill K., Smith T.J., Nottingham R., Stone N.E., Sobek C.J., Cocking J.H., Fernández R., Caballero P.A. (2017). Differentiating botulinum neurotoxin-producing clostridia with a simple, multiplex PCR assay. Appl. Environ. Microbiol..

[134] Kammerer R.A., Benoit R.M. (2014). Botulinum neurotoxins: New questions arising from structural biology. Trends Biochem. Sci..

